# Supplementary material for: Enterobacteriaceae as a Key Indicator of Huanglongbing Infection in Diaphorina citri
Source: Int J Mol Sci. 2024 May 9;25(10):5136. doi: 10.3390/ijms25105136 (PMC11120679; doi:10.3390/ijms25105136)

Supplemental Information for:

Enterobacteriaceae as a Key Indicator of Huanglongbing Infection in *Diaphorina citri*

Xing-Zhi Duan, Guo-Sen Guo, Ling-Fei Zhou, Le Li, Ze-Min Liu, Cheng Chen, Bin-Hua Wang, Lan Wu*

School of Life Science, Nanchang University, Nanchang 330022, China

*Corresponding author, mail: ncusk724@hotmail.com

Table S1 PCR Primers in this study

Primer	Primer sequence	Length (bp)
27F	AGAGTTTGATCATGGCTCAG	1465
1492R	TACGGYTACCTTGTTACGACTT	
OI1	GCGCGTATGCAATACGAGCGGCA	1160
OI2	GCCTCGCGACTTCGCAACCCAT	
338F	ACTCCTACGGGAGGCAGCAG	468
806R	GGACTACHVGGGTWTCTAAT	

Figure S1 Relationship between the abundance of ASVs from CLas (vertical axis) and Enterobacteriaceae (horizontal axis). *, $P < 0.5$; ***, $P < 0.001$.

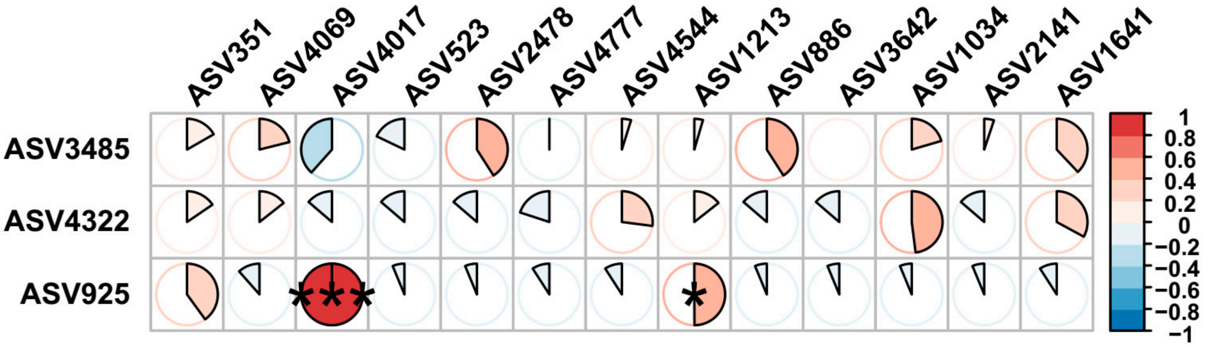

Supplement: Supplementary file 1 [file ijms-25-05136-s001.zip › ijms-2970724-supplementary.pdf]
